# The Impact of Rapid Handpump Repairs on Diarrhea Morbidity in Children: Cross-Sectional Study in Kwale County, Kenya

**DOI:** 10.2196/42462

**Published:** 2024-01-16

**Authors:** Patrick Thomson, Justin Stoler, Michelle Byford, David J Bradley

**Affiliations:** 1 School of Geography and the Environment University of Oxford Oxford United Kingdom; 2 Department of Engineering Science University of Oxford Oxford United Kingdom; 3 Department of Geography and Sustainable Development University of Miami Coral Gables, FL United States; 4 Department of Public Health Sciences University of Miami Miami, FL United States; 5 Barts Health NHS Trust London United Kingdom; 6 London School of Hygiene & Tropical Medicine London United Kingdom

**Keywords:** water, Kenya, WASH, water, sanitation, and hygiene, maintenance, diarrhea, diarrhoea, SDG 6, service provision, handpump, child health

## Abstract

**Background:**

Handpumps are used by millions of people as their main source of water. Although handpumps represent only a basic form of water provision, there have been continuous efforts to improve the performance of these systems as they are likely to remain in use for many years to come. The introduction of a professional maintenance service in southern Kenya has shown an order of magnitude improvement in operational performance over community-based management, with 90% of handpump faults repaired within 3 days of being reported. One driver behind these efforts is the assumption that a more reliable water supply will lead to a reduction in water-related disease. However, it is not clear if operational improvements lead to health gains. Despite limited empirical evidence, some modeling studies suggest that even short periods of drinking contaminated water can lead to disproportionate negative health impacts.

**Objective:**

The aim of this study was to assess whether the improvements in operational performance from the rapid professional maintenance of rural handpumps lead to improved household health outcomes.

**Methods:**

From a sample of households using handpumps as their primary water source in Kwale County, Kenya, we measured the 2-week prevalence of World Health Organization–defined diarrhea in children, reported by the adult respondent for each household. We compared the rates before and after a period during which the households’ handpumps were being professionally maintained. We then conducted a cross-sectional analysis, fitting logistic regression models with reported diarrhea as the dependent variable and speed of repair as the independent exposure of interest, adjusting for household socioeconomic characteristics; dwelling construction; and Water, Sanitation, and Hygiene (WASH)-related factors. We fitted an additional model to examine select interactions between covariates.

**Results:**

Reported diarrhea in children was lower in households whose pumps had been repaired within 24 hours (adjusted odds ratio 0.35, 95% CI 0.24-0.51). This effect was robust to the inclusion of multiple categories of covariates. No reduction was seen in households whose pump repairs took more than 24 hours. Analysis of interaction terms showed that certain interventions associated with improved WASH outcomes were only associated with reductions in diarrhea in conjunction with socioeconomic improvements.

**Conclusions:**

Only pump repairs consistently made within 24 hours of failure led to a reduction in diarrhea in the children of families using handpumps. While the efficacy of reduction in diarrhea is substantial, the operational challenges of guaranteeing same-day repairs limits the effectiveness of even best-in-class pump maintenance. Maintenance regimes that cannot bring handpump downtimes close to zero will struggle to generate health benefits. Other factors that reduce diarrhea prevalence have limited effect in isolation, suggesting that WASH interventions will be more effective when undertaken as part of more holistic poverty-reduction efforts.

## Introduction

In rural areas with no grid electricity or limited funds to run diesel generators, many communities depend upon handpumps to access shallow groundwater for their daily water needs. Groundwater, in contrast to surface water, is more likely to be available year-round and less likely to require treatment to be potable [1]. Despite efforts throughout the period of the Millennium Development Goals and now in response to Sustainable Development Goal (SDG) 6.1, “safely managed water for all,” millions of people will still be relying on water from handpumps beyond 2030. While “safely managed” water—as defined by the World Health Organization (WHO)/United Nations Children’s Fund (UNICEF) Joint Monitoring Programme (JMP) as the use of “an improved drinking water source which is located on premises, available when needed, and free of fecal and priority chemical contamination”—remains the goal for water services for all, the size of the task and difficulty in achieving this goal has been acknowledged through the JMP Service Ladder’s inclusion of an interim service level of “basic” water, the category that includes most handpumps [2].

There are many reasons for aspiring to the goal of safely managed water for all. One of the main drivers of this goal—and Water, Sanitation, and Hygiene (WASH) interventions more generally—has been the understanding that disease morbidity can be reduced through better water service provision [3]. Piped water to the home, the most usual conception of safely managed water, is associated with lower morbidity, in particular of diarrheal disease [4]. However, with 26% of the world’s population—and 73% in sub-Saharan Africa—still lacking safely managed water and the achievement of SDG 6.1 unlikely at current rates of progress [5], it is important to understand the health implications of having only a basic water supply, as these are at risk of contamination [6], which can lead to diarrheal disease [7].

The contribution of diarrheal disease to the worldwide disease burden is falling but remains substantial [8]. Diarrhea itself contributes to the burden of disease for individuals and consequently to the global burden of disease estimates. Diarrhea is the cause of 1.31 million deaths worldwide, with 303,045 of those being of children under 5 years of age in sub-Saharan Africa; for that same demographic, diarrhea also causes 27 million disability-adjusted life years (DALYs) [9], which corresponds to approximately one in every seven DALYs. Diarrhea interacts with nutrition, reducing nutrient absorption [10,11]; therefore, this figure may significantly underestimate the disease burden from diarrhea by up to 39% [12]. Again, this is especially egregious in children, as undernutrition can produce long-term, nonrecoverable negative impacts on physical and cognitive development [13-16], with associated long-term economic and welfare impacts and related policy implications [17,18].

Community-based management, the model by which local communities are responsible for the repair and maintenance of their pumps, became the default management model for rural water supply in the late 1970s, with mixed success [19-24], and an estimated one in four handpumps in sub-Saharan Africa are out of action today [25]. Since the 2000s, there has been increasing interest in professional management models more akin to how urban piped water systems are run, whether directly by governments or subcontracted to a water service provider [26-29]. More recent evidence indicates that communities do have a preference for private sector involvement in service provision over community management or direct government provision, but only if service performance is high [30-32].

We here present results obtained from a cross-sectional survey following the introduction of a professional handpump maintenance service in Kwale County in southern Kenya. The service was initially funded by a research program that was investigating whether the intervention of a rapid, professional repair service, supported by data measuring handpump use using sensors attached to the pumps [33], could result in repairing handpumps faster than had been the case under community-based management. The rapid repair service reduced average pump downtimes from approximately 1 month to a few days, an improvement that is valued by households and communities [34-37]. This study examined whether this improvement in service performance also led to health benefits. Although there is limited empirical evidence to inform service decisions, some studies suggest that even the shortest periods of drinking contaminated drinking water can lead to disproportionate negative health impacts [38,39], implying a threshold effect with a nonlinear relationship between quality of service and the health benefits of that service. Obtaining empirical evidence about this relationship would inform both local operational decisions and wider WASH policy, and in doing so shed light on the role of handpumps in meeting SDG 6.1.

We hypothesized that shorter pump downtimes resulting from the professional maintenance service would be associated with lower rates of child diarrhea. We examined this by combining data on pump failures and subsequent repairs, generated by the pump mechanics, with self-reported child diarrhea data from households that were surveyed before the repair service was introduced and after 18 months of the service. To overcome the challenges associated with an observational study, we analyzed the data using two complementary techniques. The first technique compared child diarrhea prevalence (during a 2-week period) in the same households before and after the repair service was introduced to control for time-invariant household characteristics. The second approach involved a cross-sectional analysis comparing households reporting diarrhea with those not reporting diarrhea, adjusting for household characteristics gathered at the same time as the self-reported diarrhea data.

## Methods

### Study Area

This study took place in Kwale County, located on the southern coast of Kenya between Mombasa and the border with Tanzania. This region covers an area of around 8300 km^2^ and has a population of 720,000 that is 82% rural, with the seventh-highest poverty rate among Kenya’s 47 counties. The study area was limited to the Msambweni and Matuga subcounties closer to the coast, covering an area of around 2200 km^2^. The coastal climate has a bimodal rainfall pattern with an average annual precipitation of 1400 mm with significant interannual variability.

**Figure 1:**
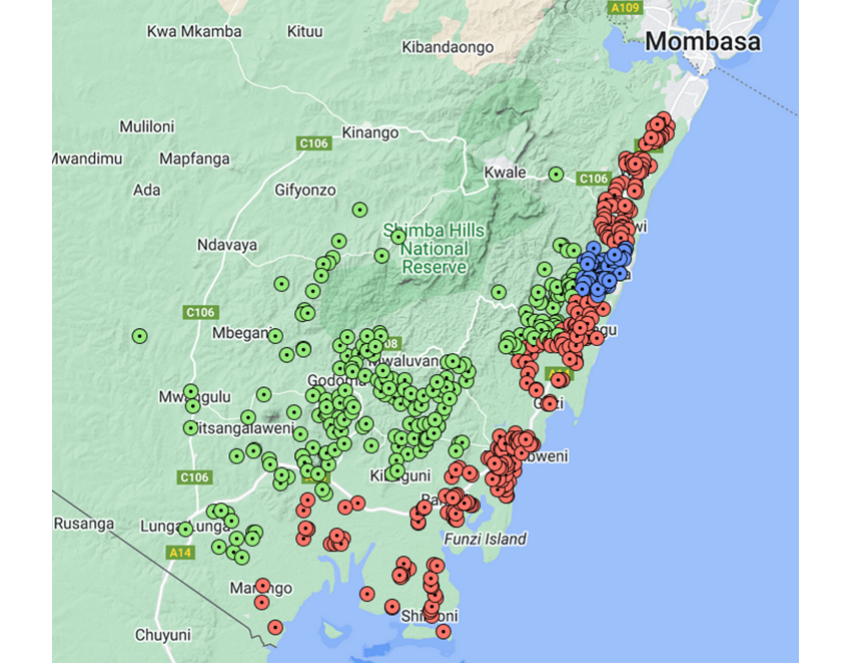
Map of the study area in Kwale County, Kenya, with the locations of handpumps marked in zones defined in Katuva et al [36]. Red indicates coastal, green indicates inland, and blue indicates Ukunda.

Within the study area there is heterogeneity both in socioeconomic characteristics and physical geography. We simplified the study area into three geographical zones for the purposes of analysis (see Figure 1) [36]. In the area in and around the urban center of Ukunda, the population density is higher and there are more alternative sources of water available during handpump breakdowns. There is also easier access to medical facilities in this area. Inland, where shallow wells are supplemented by deeper boreholes drawing water from sandstone, the population is less dense and livestock ownership is more common. Being more sparsely populated makes access to other sources of water more challenging; however, the deeper boreholes into the sandstone enable better source protection relative to shallow wells and improve the likelihood of a given handpump producing uncontaminated water. This is in contrast to the coastal strip where the population is still rural but the density is higher. Alternative nonsurface water sources in the form of other handpumps and shallow wells are relatively close by. However, this proximity of other sources, combined with the high population density and the fact that the wells in this area are mostly shallow and were hand-dug into the karstic coral, make source protection extremely difficult in this area.

### Handpump Repair Service

The professional repair service was set up in Kwale as part of a project examining whether professional handpump maintenance, aided by better data on handpumps, could lead to reduced pump downtimes [33,40]. A waterpoint mapping exercise was undertaken in August 2013, recording the location and functionality of the handpumps in the study area, along with basic information about pump repair and management arrangements. A total of 626 pumps were found, with 351 identified as having been functional during the previous 12 months. We then set up an office in Msambweni, near the center of the study area, and recruited two experienced pump fundis (mechanics) and a manger. Two hundred handpumps were offered a free maintenance service, which corresponded to all of the functioning handpumps in a smaller geographical area across Msambweni and Matuga subcounties. The service started in February 2014 and continued until the end of 2015.

We provided the mechanics with motorbikes, tools, and a stock of spare parts to be able to quickly respond to handpump failures and complete most repairs during a single visit to the pump. When repairs required more labor, such as removing rising mains, the communities provided pro-bono support to the mechanics. Toward the end of 2015, we spun the repair service out from the research program, establishing it as an independent locally owned entity, named FundiFix. At this point, communities were given the opportunity to continue receiving the maintenance service through paying a heavily subsidized monthly fee [41].

### Data Sources

**Figure 2:**
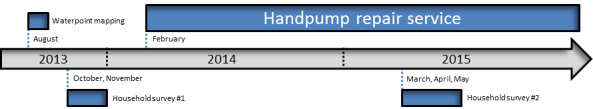
Timeline of activities that generated data used in this study.

The study timeline is schematically summarized in Figure 2. Building on the mapping exercise completed in August 2013, a more extensive household survey was conducted in October and November 2013. A total of 2508 households who had access to a functioning handpump were surveyed, randomly selected from those using each of the 351 functional handpumps identified during the earlier waterpoint mapping exercise. Household selection was made by the enumerator team leader, selecting a direction and distance from the pump for each enumerator based on a throw of a six-sided die. A mean of 7.1 households were interviewed per handpump.

The survey questionnaire included questions on household makeup and demographics; socioeconomic status, including key consumption and wealth indicators; basic self-reported health indicators for each household member; water use, collection, and storage; and waterpoint institutional arrangements, payment policies, and behavior. These questions were asked of one respondent for each household. For this study, we used the presence or absence of diarrhea reported for children in the household in the 2 weeks prior to the survey as the primary outcome measure; the other items were considered as covariates in the analysis. The only variable used from the 2013 survey was self-reported diarrhea in the 2 weeks prior to the survey.

A second household survey was conducted in March, April, and May of 2015. This was a repeat of the first survey, the aim being to see what had changed for the communities in the study area during the time of the project, specifically in relation to the pump repair service. The two surveys were matched: 59% were matched by household and respondent, 30% were matched by household but not respondent, and the remaining 11% represent cases where a different family/household was living in the dwelling. All the socioeconomic data used in this study were from the 2015 survey as these data represented the households’ situation during the time they were receiving the free repair service. At the time of this survey, the maintenance service had been operational for at least 12 months.

The two mechanics and their manager who were providing the handpump maintenance service kept detailed records of repairs undertaken, the most significant details being when the breakdown was first identified, when they attended, and when the repair was completed. These records yielded the time and duration of pump outages. Over the whole period, 599 repairs were completed on 169 pumps. The time between a breakdown being reported and repaired varied due to factors such as the complexity of the required repair, the geographical remoteness of the pump, and other logistical or operational constraints. The majority of repairs (90%) were completed within 3 days of a breakdown—the stated target of the service—and 44% were repaired within 24 hours of the fault being reported.

### Blinding and Concealment

Efforts were made to ensure blinding and concealment when collecting the data. The enumerators were blinded to the intervention as, other than being aware that the mechanics were operating in the area as part of another University of Oxford–funded research project, they had no knowledge of which pumps had been repaired. While the households were certainly not blinded to whether or not they had had a repair, it would not have been readily apparent to participants that the questions about their household members’ health were to be linked to the repairs, because the health questions were part of a large questionnaire and questions about the pump were intentionally asked in a later section of the survey than the health questions.

Mirroring the enumerators, the mechanics had very little knowledge of the household surveys. They were aware that the first household survey had taken place and that the second was occurring, and that it was broadly connected to their work, but they were not in any way involved in the survey. Treatment allocation—in this case, pump repair by the mechanics—could not be entirely randomized given that chance and other factors, measurable or otherwise, will have led to pump breakdown; however, the mechanics’ responses to reported breakdowns were unpredictable and not influenced by the enumerators or research team.

### Inclusion Criteria

A total of 1451 households used pumps that were offered the free repair service. Out of these, 294 were excluded as our mechanics had not made a repair to their handpumps during the trial period. A further 182 were excluded as it was reported during the household survey that the reference handpump was not their primary water source. Finally, 135 were excluded as there were no children reported in the household, leaving a sample of 840 households served by 139 pumps. These pumps received 412 repairs, of which 41% were repaired within 1 day and 39% more within 2 days. The longest repair time was 11 days. In the self-controlled analysis, an additional 89 households were excluded because the families living in the dwelling had changed between the two surveys.

### Statistical Analysis

Using the data from the second household survey, we first performed bivariate analysis to test the relationship between speed of pump repairs and self-reported diarrhea. We then used the household-matched data from the first survey to compare the period prevalence of diarrhea reported in 2013 against repair performance measured in 2015. This was to determine whether any relationship between the outcome and exposure was due to time-invariant characteristics of the household that were somehow correlated to the speed of repairs and not due to the repair service itself.

We then performed a cross-sectional analysis, fitting multivariable logistic regression models of self-reported diarrhea using the data from the 2015 household survey to adjust for covariates that have been shown to affect diarrhea period prevalence in the WASH-related literature. We fitted a series of multivariable regression models that iteratively adjust for an additional thematic block of covariates: (1) socioeconomic factors, (2) dwelling factors, and (3) WASH factors (see the Results for detailed factors). Additionally, we fitted a model with interaction terms to further explore results of the first three models, which was also guided by the WASH-related literature. Standard errors were adjusted for clustering using the three geographical zones to control for potentially unobserved/unquantified factors that are hypothesized to be correlated among households within each zone [42]. Because multiple households typically share a handpump, the handpump was another potential analytical unit for the clustering of standard errors between households. As a robustness check, we performed an additional analysis that clustered errors by pump instead of geographical zone.

All statistical analyses were undertaken with Stata version 14.2 (StataCorp, College Station, TX, USA).

### Ethical Considerations

This research was conducted with permission from Kenya’s National Council for Science and Technology and with ethics approval from the University of Oxford’s Central University Research Ethics Committee (CUREC reference: SOGE C1 13-125). Informed consent was given by all research participants, all of whom were aged 18 years or over.

## Results

### Unadjusted Analysis

Bivariate logistic regression models did not provide evidence of any association between households reporting diarrhea and the number of repairs performed on their respective handpumps or the time elapsed since the most recent repair. However, this analysis did reveal an association between the speed of repairs (or pump downtime) and reported diarrhea (Table 1). Pumps consistently repaired within 24 hours upon a breakdown, irrespective of the number of repairs, were associated with a lower reported diarrhea rate. This effect disappeared when analyzed against longer repair times (Table 2).

The analysis comparing the same exposure of rapid repairs in 2015 but with the outcome of self-reported disease from the same household in 2013 (ie, when the repair service could not have had any influence) showed no effect. This suggests that the disease reduction was not due to unobserved time-invariant household characteristics that were somehow correlated with the speed of repairs. Rates of reported diarrhea were comparable between the two surveys: 8.2% in 2013 (dry season) and 9.8% in 2015 (wet season).

**Table 1:** Bivariate logistic regression analysis of child diarrhea with number and speed of repairs.

Intervention/exposure		Unadjusted odds ratio (95% CI)	Analysis type
Number of repairs (2015)		0.95 (0.85-1.07)	Cross-sectional
Days since last repair (2015)		1.00 (1.00-1.00)	Cross-sectional
**Pump repairs within 24 hours**			
	2015	0.40 (0.27-0.58)	Cross-sectional
	2013	1.01 (0.44-2.29)	Self-controlled

**Table 2:** Relative risk of households reporting child diarrhea for different repair speeds.

Intervention/exposure	Number of households	Households reporting diarrhea, n (%)	Rate ratio	*P* value
All pump repairs completed within 24 hours	123	7 (5.7)	reference	N/A^a^
All repairs completed within 48 hours (with some over 24 hours)	171	23 (13.5)	2.36	.02
Repairs taking over 48 hours to complete	546	69 (12.6)	2.22	.02

^a^N/A: not applicable.

### Cross-Sectional Analysis

The cross-sectional analysis revealed possible predictors of reporting diarrhea. Table 3 shows the odds ratios for diarrhea against other household information gathered during the 2015 survey, along with summary statistics for those characteristics. The quality of dwelling construction was associated with lower reported diarrhea in children, as were having a female head of household and cultivating crops. Apparent risk factors were those related to higher household occupancy and having no regular income. The WASH-related factors of higher per-person water collection and having an improved toilet were apparently protective. Of note, 1.5 jerrycans of water were collected for each household member per day, corresponding to 30-35 liters per person, with few households treating water either by adding chlorine (eg, Waterguard) or by boiling.

**Table 3:** Bivariate logistic regression analysis of associations of household characteristics with reporting child diarrhea (12% of 840 households) from the survey conducted in March-May 2015.

Household characteristic	Unadjusted odds ratio (95% CI)	*P* value	Percentage or mean
Number of repairs	0.95 (0.85-1.07)	.42	2.7
Repairs within 24 hours	0.40 (0.27-0.58)	<.001	15%
Improved floor	0.37 (0.22-0.61)	<.001	35%
Improved roof	0.53 (0.31-0.89)	.02	34%
Improved walls	0.52 (0.39-0.71)	<.001	46%
Improved toilet	0.56 (0.38-0.81)	.002	55%
Female head	0.65 (0.46-0.92)	.01	39%
Primary religion Islam	1.03 (0.71-1.49)	.14	85%
Household head completed secondary school	0.96 (0.56-1.63)	.87	30%
Number of people per household	1.11 (1.09-1.13)	<.001	5.4
Sleeping rooms in dwelling	0.91 (0.87-0.95)	<.001	3.1
Number of people per bedroom	1.27 (1.26-1.29)	<.001	2.0
No regular income	1.41 (1.18-1.69)	<.001	43%
Own animals	1.08 (0.57-2.06)	.81	35%
Grows crops	0.89 (0.67-1.18)	.42	74%
Distance from handpump (m)	1.00 (1.00-1.00)	.64	136
Distance from health dispensary (km)	0.96 (0.84-1.10)	.57	1.8
Distance from nearest market (km)	1.05 (1.03-1.06)	<.001	5.0
Soap observed	0.65 (0.36-1.19)	.17	75%
Water treated	0.87 (0.38-1.98)	.73	10%
Water storage in the dry season (days)	1.10 (0.96-1.27)	.17	1.8
Water storage in the wet season (days)	1.02 (0.91-1.15)	.69	2.7
Number of jerrycans per person per day	0.68 (0.64-0.72)	.001	1.53

### Multivariable Analysis of Self-Reported Diarrhea

Table 4 presents the adjusted odd ratios (AORs) and 95% CIs of iterative mixed-effects multivariable logistic regression models of child diarrhea: model 1 begins with socioeconomic factors, model 2 introduces a block of dwelling-related factors, and then model 3 introduces a block of WASH-related factors. The protective effect of handpump repairs consistently made within 24 hours remained robust across all multivariable models. Of the socioeconomic factors, having a female head of household (AOR 0.54, 95% CI 0.36-0.82; *P*=.004) and growing crops (AOR 0.67, 95% CI 0.48-0.95; *P*=.02) were associated with lower reported diarrhea across all models. Having no income (AOR 1.36, 95% CI 1.11-1.69; *P*=.003) and being further from the market (AOR 1.10, 95% CI 1.07-1.13; *P*<.001) were associated with higher odds of child diarrhea. Having more people in the household was also associated with reporting diarrhea (AOR 1.12, 95% CI 1.04-1.21; *P*=.003), although this was possibly an artifact of aggregating all cases of child diarrhea in the household into a binary variable; nevertheless, an alternate analysis with the number of *children* in the household produced a similar result. Among the WASH-related factors, having an improved toilet was associated with lower diarrhea (AOR 0.61, 95% CI 0.44-0.84; *P*=.002). The effect of the dwelling characteristics was less consistent across the three models. As a robustness check, we also refitted these models with errors clustered by pump, rather than by geographical zone, but we observed no substantive differences from the results presented in Table 4.

We built on the full model (model 3) by introducing interaction terms between the following pairs of covariates: having no income and growing crops, a female household head and the presence of soap, and having an improved floor and improved toilet. These interactions are presented in Table 5. Having no regular income and not growing crops was the largest risk factor for reporting diarrhea. Strikingly, if a household was growing crops, it had no additional odds of reporting diarrhea if it had no regular income. Conversely, the benefit of having soap in a household seemed only to accrue to households headed by a woman, in which case the odds of reporting diarrhea reduced substantially. Finally, having an improved floor substantially reduced the odds of reporting diarrhea in households with an improved toilet, but provided no added benefit to those without.

**Table 4:** Mixed-effects logistic regression models of self-reported diarrhea, adjusted for blocks of covariates, using 2015 household survey data and handpump repair data from 2014 and 2015.

Characteristic		Adjusted odds ratio (95% CI)		
		Model 1	Model 2	Model 3
Repairs within 24 hours		0.38 (0.24-0.62)	0.38 (0.23-0.62)	0.34 (0.22-0.52)
**Socioeconomic factors**				
	No regular income	1.34 (1.12-1.60)	1.34 (1.11-1.61)	1.36 (1.11-1.69)
	Grows crops	0.73 (0.50-1.07)	0.67 (0.45-0.98)	0.67 (0.48-0.95)
	People per household	1.14 (1.12-1.26)	1.15 (1.09-1.20)	1.12 (1.04-1.21)
	Female head	0.63 (0.45-0.89)	0.58 (0.37-0.91)	0.54 (0.36-0.82)
	Distance from market (km)	1.06 (1.04-1.09)	1.07 (1.03-1.10)	1.10 (1.07-1.13)
**Dwelling factors**				
	Improved roof	—^a^	0.65 (0.32-1.30)	0.74 (0.38-1.44)
	Improved walls	—	0.99 (0.76-1.29)	0.99 (0.69-1.42)
	Improved floor	—	0.42 (0.21-0.85)	0.50 (0.22-1.14)
	Number of bedrooms	—	1.03 (0.99-1.07)	1.07 (1.04-1.09)
**WASH^b^ factors**				
	Improved toilet	—	—	0.61 (0.44-0.84)
	Soap observed	—	—	0.59 (0.32-1.08)
	Jerrycans per person per day	—	—	0.86 (0.69-1.07)
	Water treated	—	—	0.98 (0.40-2.41)

^a^Not included in model.

^b^WASH: Water, Sanitation, and Hygiene.

**Table 5:** Mixed-effects logistic regression model of self-reported diarrhea with interactions, using 2015 household survey data and handpump repair data from 2014 and 2015.

Characteristic			Odds ratio (95% CI)	*P* value
**Main effects**				
	Repairs within 24 hours		0.36 (0.24-0.55)	<.001
	Distance from market (km)		1.09 (1.06-1.13)	<.001
	Improved roof		0.73 (0.36-1.48)	.32
	Improved walls		0.96 (0.69-1.33)	.92
	People per household		1.11 (1.03-1.20)	.02
	Number of bedrooms		1.06 (1.03-1.10)	.02
	Jerrycans per person per day		0.85 (0.69-1.05)	.12
	Water treated		1.01 (0.42-2.46)	.98
**Interactions**				
	**Income and crops**			
		Regular income and grows crops	1.00 (0.69-1.45)	.86
		No regular income and grows crops	1.10 (0.68-1.79)	.63
		No regular income and does NOT grow crops	2.30 (1.85-2.86)	<.001
	**Household head and soap**			
		Male head and soap observed	0.79 (0.43-1.44)	.40
		Female head and soap NOT observed	0.89 (0.62-1.30)	.41
		Female head and soap observed	0.32 (0.26-0.38)	<.001
	**Standard of floor and toilet**			
		Improved floor and NO improved toilet	0.80 (0.33-1.91)	.51
		Unimproved floor and improved toilet	0.72 (0.53-0.98)	.03
		Improved floor and improved toilet	0.29 (0.13-0.65)	<.001

## Discussion

### Principal Findings

This study revealed an association between the speed of pump repairs and child diarrhea morbidity. Only households whose pumps were consistently repaired within 24 hours of a failure reported a significant reduction in child diarrhea. This finding is consistent with modeling work that suggests that even the briefest time without high-quality water can lead to disproportionate health impacts [38,39], but the microbiological pathway very likely does not fully explain the higher likelihood of self-reported illness for pumps that were repaired less rapidly. The lag time between pump failure and survey completion varied considerably, ranging from the day before the survey took place to pumps that were repaired when the maintenance service started over 1 year before the survey look place and had remained functional ever since. In these latter cases, direct microbiological causality between pump downtime and illness is unlikely.

In lieu of the presence of a consistent microbiological pathway, we would propose an additional behavioral explanation. If a household has higher confidence in the reliability of their pump, knowing that mechanical failures will be consistently fixed within 24 hours, they may be less likely to immediately fall back on using lower-quality alternative water sources as they might in the case of an extended pump downtime. If households experience any variability in the speed of pump repairs, then they are more likely to collect, store, and use water from less wholesome alternative sources. These are unlikely to be discarded once the pump is fixed, because there was a time or financial cost in acquiring them, thus raising the potential for ongoing waterborne disease exposure.

In addition to providing evidence that only rapidly repaired handpumps lead to a reduction in child diarrhea, this study highlights other factors that contribute to diarrheal disease in rural households reliant on basic water services. Striking, but unsurprising, are the socioeconomic effects, consistent with the WASH literature: the poorer you are, the more likely you are to experience water insecurity and consequently an increased burden of water-related diseases. Growing crops mitigated the impact of having no income; however, we do not have sufficient information from this study to attribute this to the nutritional benefit of growing food or because it generated occasional income or barter opportunities, either being plausible.

Individual components of traditional WASH interventions and programs did not seem to provide a beneficial health effect when considered in isolation. Our use of interaction terms (Table 5) suggested that soap being available and observed at a home was only associated with a reduction in diarrhea in households headed by women, suggesting that hygiene behaviors in children may be better taught or enforced in households headed by women than by men. Similarly, the benefits of having an improved toilet were magnified by having an improved floor (ie, one made of cement or concrete). Such floors are easier to clean, reducing the likelihood of having human and animal feces present in homes. These empirical data are consistent with conceptual models that articulate a nonlinear relationship between the reduction in fecal contamination and consequent reduction in diarrhea [43,44].

The adaptation strategy of collecting and drinking more unsafe water in response to pump unreliability increases the risk of water-related illnesses. However, this strategy is one that households will rationally stick to unless there is compelling and enduring evidence that it is not necessary. This is consistent with studies on household preferences for rapid pump maintenance [31,34], and was supported anecdotally by conversations with people who were receiving the free repair service. However, even the well-resourced maintenance service linked to this study only fixed 15% of pumps within 24 hours *every* time they broke down. Acknowledging the possible selection bias in this study, community-managed handpumps are even less likely to be *consistently* repaired within 24 hours. This is not to discount the many other benefits from reducing pump downtimes, it is simply that health cannot be assumed to be one of them.

From an operational perspective, any pump maintenance service that reduces downtimes by an order of magnitude would be viewed as excellent in the rural water sector or even a best-in-class option. This study demonstrated an association between consistent, rapid handpump repairs and reduced child diarrhea reported in the households that use these pumps, suggesting that such a high level of service may also lead to better health outcomes. Any WASH intervention that achieves a reduction in diarrhea of over 50% would certainly be viewed as a success. If professionalizing handpump operations and maintenance to minimize pump downtimes can yield the health impacts demonstrated in this small study, can it have a substantial impact on the burden of disease?

Only the pumps with the lowest downtimes were associated with this level of health benefit. The 60% reduction in diarrhea was only the best possible outcome for a best-in-class service. The households using handpumps that had received very rapid repair corresponded to only the 15% of pumps where all repairs were completed within 24 hours. An efficacy of 60% translated into an effectiveness in reducing diarrhea morbidity of less than 10% for all the households receiving the repair service. To produce a health benefit, continuity of service must be maintained with near-zero downtime. This level of service is extremely difficult for professional service providers to maintain, and there is scant evidence that community maintenance can consistently achieve these performance levels [24,25,45,46]. FundiFix now operates a hybrid system of reactive and preventative maintenance to maintain pump uptime. Work is underway to use the sensors on the handpumps to monitor the pumps’ condition and predict failures with the aim of eliminating pump downtime altogether [40,47].

### Limitations

This study’s primary outcome variable was self-reported diarrhea, with enumerators trained to ask the question according to the WHO definition. Self-reporting is known to have limitations, and diarrhea is a crude proxy for water-related diseases [48,49]. The use of self-reported diarrhea in this study does not in itself call into question the primary finding that consistently rapid repairs are paramount to the delivery of health benefits, but it does mean that caution should be used in interpreting the effect size measured here. As the cross-sectional element of the study was based around one survey, the primary outcome variable was measured at times that varied with respect to the primary exposure. This had advantages for blinding and concealment, but limits the ability to attribute any observed effect to a specific breakdown event. We can only speculate on the extent to which the mechanism for the observed effect is directly biological or behavioral.

Household matching between the two surveys was undertaken rigorously, with unmatched households excluded from analysis where appropriate. The two surveys were 18 months apart and the household characteristics were not necessarily identical during each survey wave. Household composition may have changed with births, deaths, or others joining or leaving the household; household water budgets may also have changed as members transitioned from child to adult roles, or from being breastfed to directly consuming food and water, and other factors.

The exclusion criteria used for this study necessarily restricted the survey to households confirmed to have used pumps that had received repairs from the free maintenance service. The average downtime for handpumps across the study area prior to the trial was 27 days. It is plausible that the communities that chose not to engage with the repair service were those with better repair arrangements and performance prior to the study, introducing a possible selection bias. In addition, the method of household selection was likely to have created a bias toward households living closer to the pump (the average distance from a dwelling to its reference handpump was 136 meters and the maximum was 739 meters). These issues do not affect the internal validity of the study but may reduce external validity and the wider inferences that can be made.

Most of these limitations were the result of using an observational study design, which was linked to establishing the local pump maintenance provider. While the findings may be less widely generalizable, opportunistic operational studies linked to local WASH programs—whether by governments, private operators, or development agencies—have certain benefits. Here, the findings are directly informing the service provider’s operational management of handpumps in this area. By operating within existing and ongoing local programs in this way, findings from such studies can feed directly into local decision-making, and are thus more likely to have an immediate and sustainable impact on local health outcomes [50]. In this case, these findings—already shared with the maintenance service provider—have underscored the importance of reducing pump downtimes to a minimum.

### Conclusions

Implicit in the JMP’s definition of “basic water” is the assumption that improved sources of water are of higher quality than unimproved sources and would therefore be associated with the health benefits of lower diarrheal disease morbidity. Our findings suggest that handpumps may only produce health benefits if service delivery can achieve the highest levels of performance, a level of performance difficult to maintain even under ideal operational circumstances. These findings provide empirical support to prior modeling of the relationship among water service reliability, water quality, and health, which suggests that even short periods of supply intermittency may have large adverse health impacts [38,39].

The required consistent reduction in downtimes needed is a challenge for even FundiFix, a well-resourced and tightly managed service provider that achieves higher uptimes than are typical in the sector. Our findings have implications beyond handpumps and are potentially relevant to other forms of *basic* water service. For these, the management arrangements are often limited, back-up sources are most likely from an unimproved source, and similar household choices will have to be made in response to an unreliable supply. This underscores the need to pursue on-premises, continuous supply.

These findings are also consistent with a rich literature linking water-related diseases to poverty. We found that socioeconomic factors were more closely linked to household disease than some classic WASH factors, which taken in isolation did not reduce the incidence of child diarrhea. This implies that WASH interventions will be more effective when integrated into wider antipoverty and service delivery efforts, rather than implemented in isolation. Both of these findings support the current direction of the WASH and Water Security research agenda, which considers the key role water plays in other fields such as education and nutrition, and emphasizes the wider systems of service delivery, governance, and rights of which WASH interventions are a part [51,52].

## Abbreviations

AOR: adjusted odds ratio
DALYs: disability-adjusted life years
JMP: Joint Monitoring Programme
SDG: Sustainable Development Goal
UNICEF: United Nations Children's Fund
WASH: Water, Sanitation, and Hygiene
WHO: World Health Organization

## Data Availability

The full data analyzed for this study are not publicly available in order to maintain the confidentiality and anonymity of the participants. The publicly available data, which were generated during UK Research and Innovation–funded projects ES/JO18120/1 and NE/M008894/1, are available in the UK Data Service repository [53].

## References

[1] MacDonald A, Calow R (2009). Developing groundwater for secure rural water supplies in Africa. Desalination.

[2] Water, Sanitation, Hygiene and Health (WSH) WHO Team (2019). Safely managed drinking water - thematic report on drinking water 2017. World Health Organization.

[3] Prüss-Ustün A, Wolf J, Bartram J, Clasen T, Cumming O, Freeman MC, Gordon B, Hunter PR, Medlicott K, Johnston R (2019). Burden of disease from inadequate water, sanitation and hygiene for selected adverse health outcomes: an updated analysis with a focus on low- and middle-income countries. Int J Hyg Environ Health.

[4] Fink G, Günther I, Hill K (2011). The effect of water and sanitation on child health: evidence from the demographic and health surveys 1986-2007. Int J Epidemiol.

[5] Water, Sanitation, Hygiene and Health (WSH) WHO Team (2021). Progress on household drinking water, sanitation and hygiene 2000‒2020: Five years into the SDGs. World Health Organization.

[6] Bain R, Cronk R, Wright J, Yang H, Slaymaker T, Bartram J (2014). Fecal contamination of drinking-water in low- and middle-income countries: a systematic review and meta-analysis. PLoS Med.

[7] Murphy H, Prioleau M, Borchardt M, Hynds P (2017). Review: Epidemiological evidence of groundwater contribution to global enteric disease, 1948–2015. Hydrogeol J.

[8] Wang H, Liddell CA, Coates MM, Mooney MD, Levitz CE, Schumacher AE, Apfel H, Iannarone M, Phillips B, Lofgren KT, Sandar L, Dorrington RE, Rakovac I, Jacobs TA, Liang X, Zhou M, Zhu J, Yang G, Wang Y, Liu S, Li Y, Ozgoren AA, Abera SF, Abubakar I, Achoki T, Adelekan A, Ademi Z, Alemu ZA, Allen PJ, AlMazroa MA, Alvarez E, Amankwaa AA, Amare AT, Ammar W, Anwari P, Cunningham SA, Asad MM, Assadi R, Banerjee A, Basu S, Bedi N, Bekele T, Bell ML, Bhutta Z, Blore JD, Basara BB, Boufous S, Breitborde N, Bruce NG, Bui LN, Carapetis JR, Cárdenas R, Carpenter DO, Caso V, Castro RE, Catalá-Lopéz F, Cavlin A, Che X, Chiang PP, Chowdhury R, Christophi CA, Chuang T, Cirillo M, da Costa Leite I, Courville KJ, Dandona L, Dandona R, Davis A, Dayama A, Deribe K, Dharmaratne SD, Dherani MK, Dilmen U, Ding EL, Edmond KM, Ermakov SP, Farzadfar F, Fereshtehnejad S, Fijabi DO, Foigt N, Forouzanfar MH, Garcia AC, Geleijnse JM, Gessner BD, Goginashvili K, Gona P, Goto A, Gouda HN, Green MA, Greenwell KF, Gugnani HC, Gupta R, Hamadeh RR, Hammami M, Harb HL, Hay S, Hedayati MT, Hosgood HD, Hoy DG, Idrisov BT, Islami F, Ismayilova S, Jha V, Jiang G, Jonas JB, Juel K, Kabagambe EK, Kazi DS, Kengne AP, Kereselidze M, Khader YS, Khalifa SEAH, Khang Y, Kim D, Kinfu Y, Kinge JM, Kokubo Y, Kosen S, Defo BK, Kumar GA, Kumar K, Kumar RB, Lai T, Lan Q, Larsson A, Lee J, Leinsalu M, Lim SS, Lipshultz SE, Logroscino G, Lotufo PA, Lunevicius R, Lyons RA, Ma S, Mahdi AA, Marzan MB, Mashal MT, Mazorodze TT, McGrath JJ, Memish ZA, Mendoza W, Mensah GA, Meretoja A, Miller TR, Mills EJ, Mohammad KA, Mokdad AH, Monasta L, Montico M, Moore AR, Moschandreas J, Msemburi WT, Mueller UO, Muszynska MM, Naghavi M, Naidoo KS, Narayan KV, Nejjari C, Ng M, de Dieu Ngirabega J, Nieuwenhuijsen MJ, Nyakarahuka L, Ohkubo T, Omer SB, Caicedo AJP, Pillay-van Wyk V, Pope D, Pourmalek F, Prabhakaran D, Rahman SU, Rana SM, Reilly RQ, Rojas-Rueda D, Ronfani L, Rushton L, Saeedi MY, Salomon JA, Sampson U, Santos IS, Sawhney M, Schmidt JC, Shakh-Nazarova M, She J, Sheikhbahaei S, Shibuya K, Shin HH, Shishani K, Shiue I, Sigfusdottir ID, Singh JA, Skirbekk V, Sliwa K, Soshnikov SS, Sposato LA, Stathopoulou VK, Stroumpoulis K, Tabb KM, Talongwa RT, Teixeira CM, Terkawi AS, Thomson AJ, Thorne-Lyman AL, Toyoshima H, Dimbuene ZT, Uwaliraye P, Uzun SB, Vasankari TJ, Vasconcelos AMN, Vlassov VV, Vollset SE, Waller S, Wan X, Weichenthal S, Weiderpass E, Weintraub RG, Westerman R, Wilkinson JD, Williams HC, Yang YC, Yentur GK, Yip P, Yonemoto N, Younis M, Yu C, Jin KY, El Sayed Zaki M, Zhu S, Vos T, Lopez AD, Murray CJL (2014). Global, regional, and national levels of neonatal, infant, and under-5 mortality during 1990-2013: a systematic analysis for the Global Burden of Disease Study 2013. Lancet.

[9] GBD Diarrhoeal Diseases Collaborators (2017). Estimates of global, regional, and national morbidity, mortality, and aetiologies of diarrhoeal diseases: a systematic analysis for the Global Burden of Disease Study 2015. Lancet Infect Dis.

[10] Lindsay J (1997). Chronic sequelae of foodborne disease. Emerg Infect Dis.

[11] Schlaudecker EP, Steinhoff MC, Moore SR (2011). Interactions of diarrhea, pneumonia, and malnutrition in childhood: recent evidence from developing countries. Curr Opin Infect Dis.

[12] Troeger C, Colombara DV, Rao PC, Khalil IA, Brown A, Brewer TG, Guerrant RL, Houpt ER, Kotloff KL, Misra K, Petri WA, Platts-Mills J, Riddle MS, Swartz SJ, Forouzanfar MH, Reiner RC, Hay SI, Mokdad AH (2018). Global disability-adjusted life-year estimates of long-term health burden and undernutrition attributable to diarrhoeal diseases in children younger than 5 years. Lancet Glob Health.

[13] Lorntz B, Soares A, Moore S, Pinkerton R, Gansneder B, Bovbjerg VE, Guyatt H, Lima AM, Guerrant RL (2006). Early childhood diarrhea predicts impaired school performance. Pediatr Infect Dis J.

[14] Crookston B, Dearden K, Alder S, Porucznik CA, Stanford JB, Merrill RM, Dickerson TT, Penny ME (2011). Impact of early and concurrent stunting on cognition. Matern Child Nutr.

[15] MacIntyre J, McTaggart J, Guerrant RL, Goldfarb DM (2014). Early childhood diarrhoeal diseases and cognition: are we missing the rest of the iceberg?. Paediatr Int Child Health.

[16] Pinkerton R, Oriá RB, Lima AAM, Rogawski ET, Oriá MOB, Patrick PD, Moore SR, Wiseman BL, Niehaus MD, Guerrant RL (2016). Early childhood diarrhea predicts cognitive delays in later childhood independently of malnutrition. Am J Trop Med Hyg.

[17] Connolly MP, Topachevskyi O, Standaert B, Ortega O, Postma M (2012). The impact of rotavirus vaccination on discounted net tax revenue in Egypt: a government perspective analysis. Pharmacoeconomics.

[18] Muangchana C, Riewpaiboon A, Jiamsiri S, Thamapornpilas P, Warinsatian P (2012). Economic analysis for evidence-based policy-making on a national immunization program: a case of rotavirus vaccine in Thailand. Vaccine.

[19] Kleemeier E (2000). The impact of participation on sustainability: an analysis of the Malawi Rural Piped Scheme Program. World Development.

[20] Isham J, Kahkonen S (2002). Institutional determinants of the impact of community-based water services: evidence from Sri Lanka and India. Econ Dev Cult Change.

[21] Mansuri G (2004). Community-based and -driven development: a critical review. The World Bank Research Observer.

[22] Prokopy LS (2005). The relationship between participation and project outcomes: evidence from rural water supply projects in India. World Development.

[23] Davis J, Iyer P, Yavuz E (2006). Rural water supply, sanitation, and hygiene: a review of 25 years of lending 1978-2003. The World Bank.

[24] Chowns E (2015). Is community management an efficient and effective model of public service delivery? Lessons from the Rural Water Supply Sector in Malawi. Public Admin Dev.

[25] Foster T, Furey S, Banks B, Willetts J (2019). Functionality of handpump water supplies: a review of data from sub-Saharan Africa and the Asia-Pacific region. Int J Water Res Dev.

[26] Kleemeier E (2010). Private operators and rural water supplies: can it work?. IRC.

[27] Carter R, Harvey E, Casey V (2010). User financing of rural handpump water services. https://www.ircwash.org/resources/user-financing-rural-handpump-water-services-paper-presented-irc-symposium-%E2%80%98-pumps-pipes.

[28] Harvey P, Reed R (2006). Community-managed water supplies in Africa: sustainable or dispensable?. Community Dev J.

[29] Thomson P, Koehler J (2016). Performance-oriented monitoring for the Water SDG – challenges, tensions and opportunities. Aquatic Procedia.

[30] Hope R, Thomson P, Koehler J, Foster T (2020). Rethinking the economics of rural water in Africa. Oxf Rev Econ Policy.

[31] Hope R (2015). Is community water management the community's choice? Implications for water and development policy in Africa. Water Policy.

[32] Koehler J, Rayner S, Katuva J, Thomson P, Hope R (2018). A cultural theory of drinking water risks, values and institutional change. Global Env Change.

[33] Thomson P, Hope R, Foster T (2012). GSM-enabled remote monitoring of rural handpumps: a proof-of-concept study. J Hydroinformatics.

[34] Hope R, Ballon P (2019). Global water policy and local payment choices in rural Africa. NPJ Clean Water.

[35] Hope R, Thomson P, Koehler J, Foster T, Thomas M (2014). From rights to results in rural water services - evidence from Kyuso, Kenya. Smith School of Enterprise and the Environment, University of Oxford.

[36] Katuva J, Hope R, Foster T, Koehler J, Thomson P (2020). Groundwater and welfare: a conceptual framework applied to coastal Kenya. Groundw Sustain Dev.

[37] Thomson P, Bradley D, Katilu A, Katuva J, Lanzoni M, Koehler J, Hope R (2019). Rainfall and groundwater use in rural Kenya. Sci Total Environ.

[38] Brown J, Clasen T (2012). High adherence is necessary to realize health gains from water quality interventions. PLoS One.

[39] Hunter PR, Zmirou-Navier D, Hartemann P (2009). Estimating the impact on health of poor reliability of drinking water interventions in developing countries. Sci Total Environ.

[40] Thomson P (2020). Remote monitoring of rural water systems: a pathway to improved performance and sustainability?. WIREs Water.

[41] Koehler J, Thomson P, Goodall S, Katuva J, Hope R (2021). Institutional pluralism and water user behavior in rural Africa. World Development.

[42] Colin Cameron A, Miller DL (2015). A practitioner’s guide to cluster-robust inference. J Hum Resour.

[43] Esrey SA, Feachem RG, Hughes JM (1985). Interventions for the control of diarrhoeal diseases among young children: improving water supplies and excreta disposal facilities. Bull World Health Organ.

[44] Robb K, Null C, Teunis P, Yakubu H, Armah G, Moe CL (2017). Assessment of fecal exposure pathways in low-income urban neighborhoods in Accra, Ghana: rationale, design, methods, and key findings of the SaniPath Study. Am J Trop Med Hyg.

[45] Nagel C, Beach J, Iribagiza C, Thomas EA (2015). Evaluating cellular instrumentation on rural handpumps to improve service delivery-a longitudinal study in rural Rwanda. Environ Sci Technol.

[46] Fisher MB, Shields KF, Chan TU, Christenson E, Cronk RD, Leker H, Samani D, Apoya P, Lutz A, Bartram J (2015). Understanding handpump sustainability: determinants of rural water source functionality in the Greater Afram Plains region of Ghana. Water Resour Res.

[47] Greeff H, Manandhar A, Thomson P, Hope R, Clifton DA (2019). Distributed inference condition monitoring system for rural infrastructure in the developing world. IEEE Sensors J.

[48] Aiemjoy K, Aragie S, Gebresillasie S, Fry DM, Dagnew A, Hailu D, Chanyalew M, Tadesse Z, Stewart A, Callahan K, Freeman M, Neuhaus J, Arnold BF, Keenan JD (2018). Defining diarrhea: a population-based validation study of caregiver-reported stool consistency in the Amhara region of Ethiopia. Am J Trop Med Hyg.

[49] Johnston B, Shamseer L, da Costa BR, Tsuyuki R, Vohra S (2010). Measurement issues in trials of pediatric acute diarrheal diseases: a systematic review. Pediatrics.

[50] Abimbola S (2021). The uses of knowledge in global health. BMJ Glob Health.

[51] Wutich A, Jepson WE, Stoler J, Thomson P, Kooy M, Brewis A, Staddon C, Meehan K (2021). A global agenda for household water security: measurement, monitoring, and management. J American Water Resour Assoc.

[52] Workman CL, Cairns MR, de Los Reyes FL, Verbyla ME (2021). Global water, sanitation, and hygiene approaches: anthropological contributions and future directions for engineering. Environ Eng Sci.

[53] Hope R, Koehler J, Katuva J, Thomson P, Goodall S, Thomas M, Foster T (2022). Longitudinal panel study data on household welfare, water resource management and governance in Kenya 2013-2016. UK Data Service ReShare.

